# A comprehensive study based on exosome-related immunosuppression genes and tumor microenvironment in hepatocellular carcinoma

**DOI:** 10.1186/s12885-022-10463-0

**Published:** 2022-12-22

**Authors:** Zhan Yang, Xinmiao Li, Chaoran Pan, Yifei Li, Lifan Lin, Yan Jin, Jianjian Zheng, Zhengping Yu

**Affiliations:** Department of Hepatobiliary Surgery, The First Affiliated Hospital of Wenzhou Medical University, No.2 fuxue lane, Wenzhou, 325000 Zhejiang China

**Keywords:** Hepatocellular carcinoma, Exosome, Immunosuppressive gene, Tumor microenvironment, Immunotherapy

## Abstract

**Background:**

Exosomes play an important role in the tumor microenvironment (TME) and the mechanisms of tumor immune escape in hepatocellular carcinoma (HCC). It is known that immunosuppressive genes, involved in the processes of tumor immunosuppression, are associated with cancer progression. This study aimed to explore the prognostic values of exosome-related immunosuppression genes (ERIGs) in HCC.

**Methods:**

The RNA-seq transcriptome data of 374 HCC patients were obtained from the Cancer Genome Atlas (TCGA) database. The TCGA cohort was randomly divided into the training cohort and validation cohort in a 1:1 ratio. WGCNA analysis and Pearson correlation analysis were used to identify ERIGs. The Lasso regression method was used to construct a 5-ERIG signature. The prognostic value of our signature was examined in the First Affiliated Hospital of Wenzhou Medical University (FAHWMU) cohort.

**Results:**

Univariate Cox regression analysis was used to screen prognostic ERIGs. Subsequently, these prognostic ERIGs were included in Lasso regression analyses to identify 5 key ERIGs (ASAP1, IARS1, GTF3C2, TPD5L2 and SLC52A2) and construct a 5-ERIG signature. The patients in the low-risk group had better prognosis than those in the high-risk group. Univariate and multivariate cox regression revealed that risk score was an independent prognostic risk factor of HCC. Gene set enrichment analysis (GSEA) showed that this signature was highly associated with TME-related pathways. Subsequent analyses revealed the potential role of the signature in regulating the TME in HCC. In addition, a lower immunotherapy score was found in patients with high risk-score. Of note, this signature was confirmed to have a good performance in predicting HCC prognosis in the FAHWMU cohort. Moreover, knockdown of 5 ERIGs of this signature contributed to the suppression the Hep3B cell proliferation.

**Conclusions:**

We generated a novel prognostic 5-ERIG signature to accurately predict the prognosis of patients with HCC, and this signature may serve as an indicator of immunotherapy for HCC.

**Supplementary Information:**

The online version contains supplementary material available at 10.1186/s12885-022-10463-0.

## Introduction

Hepatocellular carcinoma (HCC) is one of the most prevalent malignancies with a high mortality rate in the world [1, 2]. In recent years, new early screening and treatment methods for HCC have been developed [3, 4]. However, the prognosis of HCC patients remains poor, with a 5-year survival rate of 12 ~ 18% [5–7]. Besides, the selection of treatment criteria and the identification of high-risk patients remain difficult due to individual heterogeneity. Therefore, it is crucial to explore potential biomarkers to predict HCC prognosis and guide individualized therapies.

Exosomes, a group of small extracellular vesicles with a diameter of 30–200 nm that can transport a variety of biomolecules between cells, have been found to play important roles in physiology and pathology of various human diseases and cancers [8–10]. In HCC, increasing studies have shown that exosomes are involved in tumor microenvironment (TME) remodeling, tumor progression, immune escape and tumor metastasis [11–13].

Tumor immune escape refers to the phenomenon that tumor cells can survive and proliferate by evading the recognition and attack of immune system through various mechanisms [14, 15]. As one of these mechanisms, tumors have been shown to produce immunosuppressive molecules to suppress immune responses [16–18]. Emerging studies have indicated that exosomes play crucial roles in tumor immunosuppression [19]. For example, tumor-derived exosomes could drive immunosuppressive macrophages to activate tumor immunosuppression [20]. Cancer-secreted exosome miR-1468-5p promotes tumor immune escape through immunosuppressive reprogramming of lymphatic vessels [21]. However, the role of exosome-related immunosuppression genes (ERIGs) in HCC development and TME regulation remains largely unknown. Therefore, this study aimed to identify the prognostic ERIGs in HCC, and explore their values in regulating tumor microenvironment, predicting the prognosis and the efficacy of immunotherapy of HCC patients.

Herein, we integrated genomic information from HCC tissues in the TCGA cohort to explore key ERIGs in HCC and their prognostic significance. On the basis of identified ERIGs, we constructed a 5-ERIG signature to evaluate HCC prognosis. Finally, we explored the potential role of the signature in regulating TME and knockdown of 5 ERIGs of this signature contributed to the suppression the Hep3B cell proliferation.

## Materials and methods

### Data acquisition

Peripheral blood exosome sequencing data from 112 HCC patients and 118 healthy individuals were obtained from exoRbase database (http://www.exorbase.org/). The RNA-seq transcriptome data of 374 HCC tissues, 50 adjacent normal tissues and corresponding clinical data were extracted from the Cancer Genome Atlas (TCGA) (http://cancergenome.nih.gov/) database, then 370 HCC patients with complete clinicopathological data were enrolled for further study. The RNA-seq transcriptome data of HCC samples in the International Cancer Genome Consortium (ICGC) (https://dcc.icgc.org/) were treated as an external validation cohort, which consisted of 231 HCC patients. In addition, a local cohort, which has a total of 100 surgically resected HCC tissues, was obtained from the First Hospital of Wenzhou Medical University (FAHWMU). The collection of this cohort was reviewed and approved by the human research ethics committee of the FAHWMU. The patients/participants provided their written informed consent to participate in this study.

### Identification of exosome genes

Genes with |log FC | > 1 and FDR < 0.05 were identified as differentially expressed genes (DEGs) using the R package “limma” in the TCGA cohort. Similarly, differentially expressed exosome-derived mRNAs were identified between HCC patients and healthy individuals (|log FC| > 0 and FDR < 0.05). Then, we took intersection of the DEGs and differentially expressed exosome-derived mRNAs to obtain differentially expressed exosomes genes in HCC.

### Dysfunction score and exclusion score

Tumor Immune Dysfunction and Exclusion (TIDE) algorithm (http://tide.dfci.harvard.edu) was applied to obtain TIDE score, including Dysfunction score and Exclusion score [22]. Then Wilcox test was used to analyze differences in dysfunction score and exclusion score between normal and tumor-tissues. The “surv-cutpoint” function was applied to obtain the best cut-ff value of Dysfunction score and Exclusion score, and then patients were divided into two groups based on the best cut-off value.

### Weighted gene co-expression network analysis (WGCNA) analysis

A total of 7684 DEGs in the TCGA cohort and exclusion score as well as dysfunction score of each sample were included in WGCNA analysis. R packets “matrixStats”, “Hmisc”, “Foreach”, “doParways”, “fast cluster”, “DynamicTreeCut”, “Survival” and “WGCNA” were applied to WGCNA analysis [23].

### Construction of a 5-ERIG prognostic signature

A total of 370 HCC patients in TCGA cohort were randomly divided into the training cohort (*n* = 185) and the validation cohort (*n* = 185) with a ratio of 1:1. Univariate Cox regression was used to screen prognostic ERIGs in the training cohort. Then, in the training cohort, these identified prognostic ERIGs were incorporated into the Lasso regression analysis to determine the vital ERIGs and construct a signature. The risk score of each HCC patient was calculated as followed formula:

$$\textrm{RiskScore}=\textrm{e}\hat{\mkern6mu} \sum \textrm{Coef}\left(\textrm{i}\right)\textrm{X}\left(\textrm{i}\right)$$

where e is the natural logarithm, Coef(i) is the coefficient, and X(i) represents the expression of ERIGs. The median of the risk score of the train cohort was used as the cut-off value to classify all HCC patients into the high- and low-risk groups.

### Validation of the prognostic signature

The Kaplan-Meier curves were used to compare the overall survival (OS) of the high- and low-risk group by the “survivor” and “survminer” R packages. Time-dependent receiver operating characteristic (ROC) curves and area under the curve (AUC) were calculated via R package “time ROC” to evaluate the accuracy of the prognostic signature. In addition, the prognostic value of risk score was verified by univariate and multivariate Cox regression analysis. A nomogram was developed using the R package “regplot” to predict the 1, 2, and 3-year OS of HCC patients. Calibration curves were plotted using R package “RMS” and “Survival” to evaluate the predictive power of this nomogram.

### Gene set enrichment analysis (GSEA)

In the TCGA cohort, differential expression analysis of all genes between the low-risk group and the high-risk group was performed using the R package “limma”. The R package “clusterProfiler” was then used for GSEA of all genes, and gene ontology (GO) was included as the gene set analyzed. The FDR < 0.05 was considered statistically significant.

### Acquisition of TME-related pathways and immunotherapy scores

The infiltrating scores of 23 types immune cells and stromal-related pathways scores were calculated via single-sample gene set enrichment analysis (ssGSEA) using the R package “gsva”. Immunotherapy scores (IPS) were downloaded from the TCGA database.

### Cell culture

The human HCC cell line Hep3B was purchased from ATCC. Hep3B was cultured in DMEM medium with 10% fetal bovine serum (FBS) and 1% antibiotics. Cells were maintained in a 37 °C incubator with 5% CO_2_.

### Cell transfection

Hep3B cells were cultured in a 6-well plate with 6 × 10^3^ cells per well. When the cell density was near to 50%, si-NC, si-ASAP1, si-IARS1, si-GTF3C2, si-TPD5L2 and si-SLC52A2 packaged by lipo2000 were transfected into cells at 37 °C for 6 h. Then fresh medium was replaced and cells were collected for subsequent experiments after 48 h of transfection.

### Quantitative real-time PCR (qRT-PCR)

Analysis Total RNA was isolated from HCC tissues using the Tiangen RNA extraction reagent kit. Each sample (1 μg RNA) was reversely transcribed into complementary DNA (cDNA) using a reverse-transcription (RT) reagent kit (Takara Biotechnology Co., Ltd., Dalian, China). Then, Real-time PCR was performed using SYBR Premix ExTaq (Takara). GAPDH were used as endogenous controls for mRNAs.

### Cell proliferation assays

Cell Counting Kit-8 (CCK8) (Dojindo, Japan) was used for the assessment of cell proliferation. Hep3B cells were seeded into 24-well plate at a density of 1 × 10^3^/ ml per well to incubate for 48 h. Then, 100 μl CCK8 solution were added to each well and maintained in a 37 °C incubator for 1 h. Finally, the absorbance of each well was measured at 450 nm.

### Statistical analysis

R version 4.1.0 (http://www.R-projectt.org) and its appropriate packages were used to perform all statistical analyses. Data are presented as mean ± SD of at least three independent experiments, and differences between two groups were compared using student’s t-test. Rank correlations were assessed by the performance of spearman’s correlation coefficient test among different variables. The R package “survival” was used for survival analysis, and Kaplan-Meier survival curves were used to display survival differences between different groups. The Log-rank test was used to assess the significance of the difference in survival time between two groups. Statistical *p*-values were subjected to two tailed tests, and *p* < 0.05 was considered as significance.

## Results

### Significant immunosuppression modules in HCC based on TIDE score

**Fig. 1:**
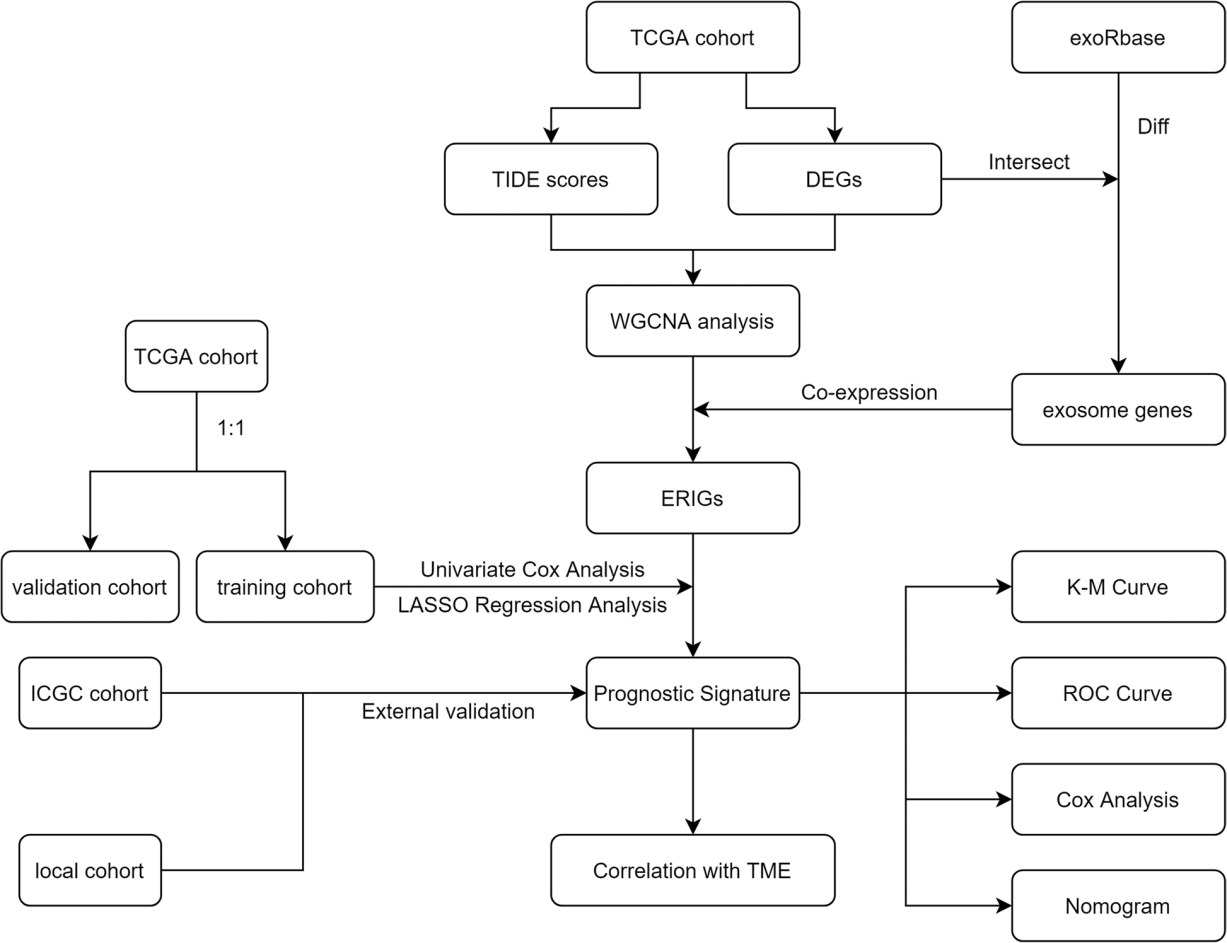
The flow chart of this study

**Fig. 2:**
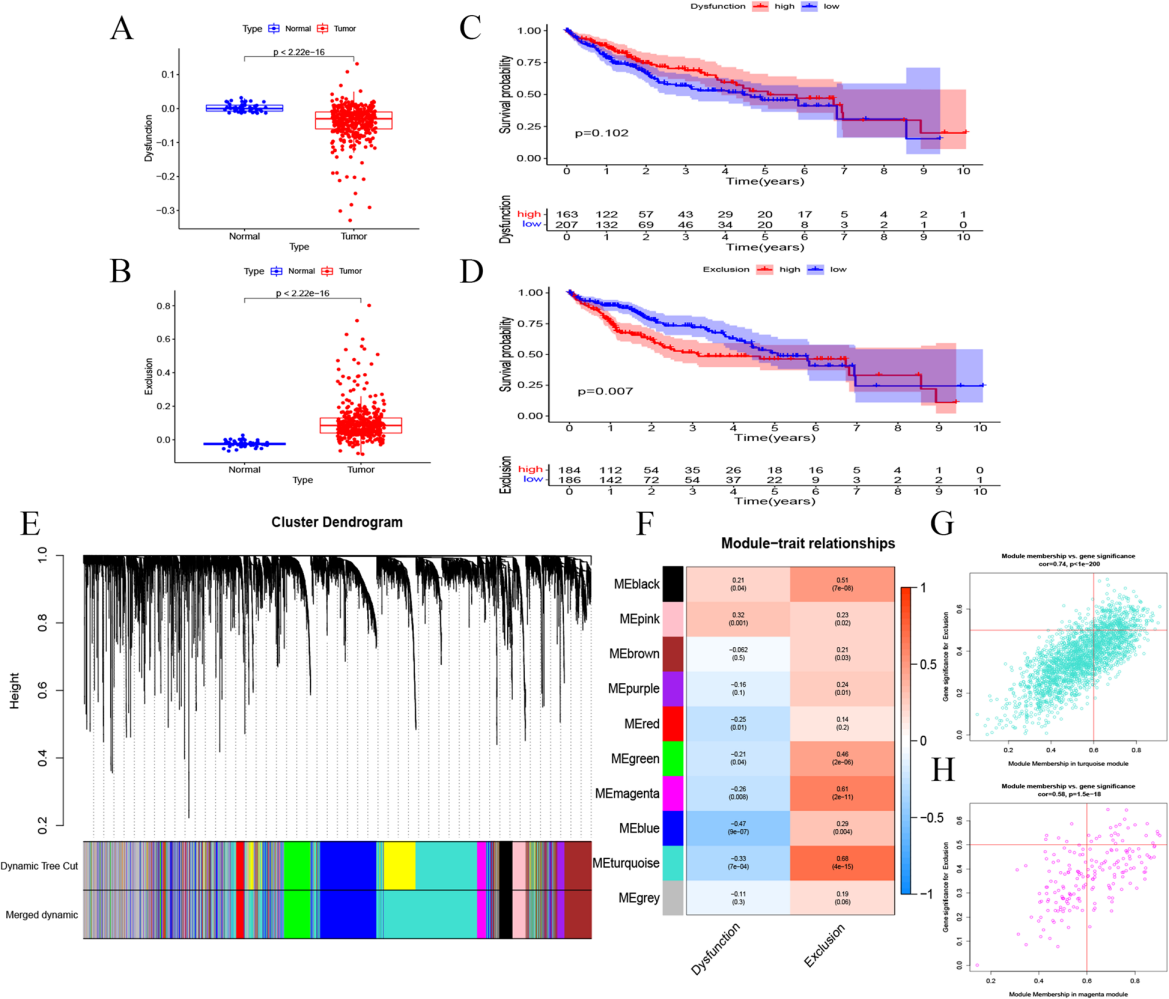
Significant immunosuppression modules in HCC based on TIDE score. **a** Expression level of Dysfunctions score in normal and tumor tissues. **b** Expression level of Exclusion score in normal and tumor tissues. **c** Kaplan-Meier survival analysis of the relationship between Dysfunction score and survival time. **d** Kaplan-Meier survival analysis of the relationship between Exclusion score and survival time. **e** WGCNA analysis of DEGs. Branches with different colors correspond to eight different modules. **f** Module-trait relationships. Scatter plot analysis of modules in the turquoise (**g**) and magenta (**h**) modules

Figure 1 showed the technical route of this study. Firstly, the differences of dysfunction and exclusion scores between normal and tumor tissues were explored. As shown in Fig. 2A, dysfunction score in normal tissues was significantly higher than that in HCC tissues (*p* < 0.05). Survival analysis showed that dysfunction score was not correlated with the prognosis of HCC patients (*p* > 0.05) (Fig. 2C). Exclusion score in HCC tissues was higher than that in normal tissues (*p* < 0.05) (Fig. 2B). In addition, the patients with high exclusion score had worse prognosis (Fig. 2D). WGCNA analysis was then applied to identify the hub gene modules, which were correlated with dysfunction score and exclusion score, on the basis of TCGA DEGs (Fig. 2E). The results revealed that the MEmagenta and MEturquoise modules were positively correlated with exclusion score (*R* > 0.6) (Fig. 2F-H). Thus, the MEmagenta and MEturquoise modules were considered as immunosuppression-related gene modules, and a total of 365 genes in the MEmagenta and MEturquoise modules were identified as immunosuppressive genes.

### Identification of prognostic ERIGs

**Fig. 3:**
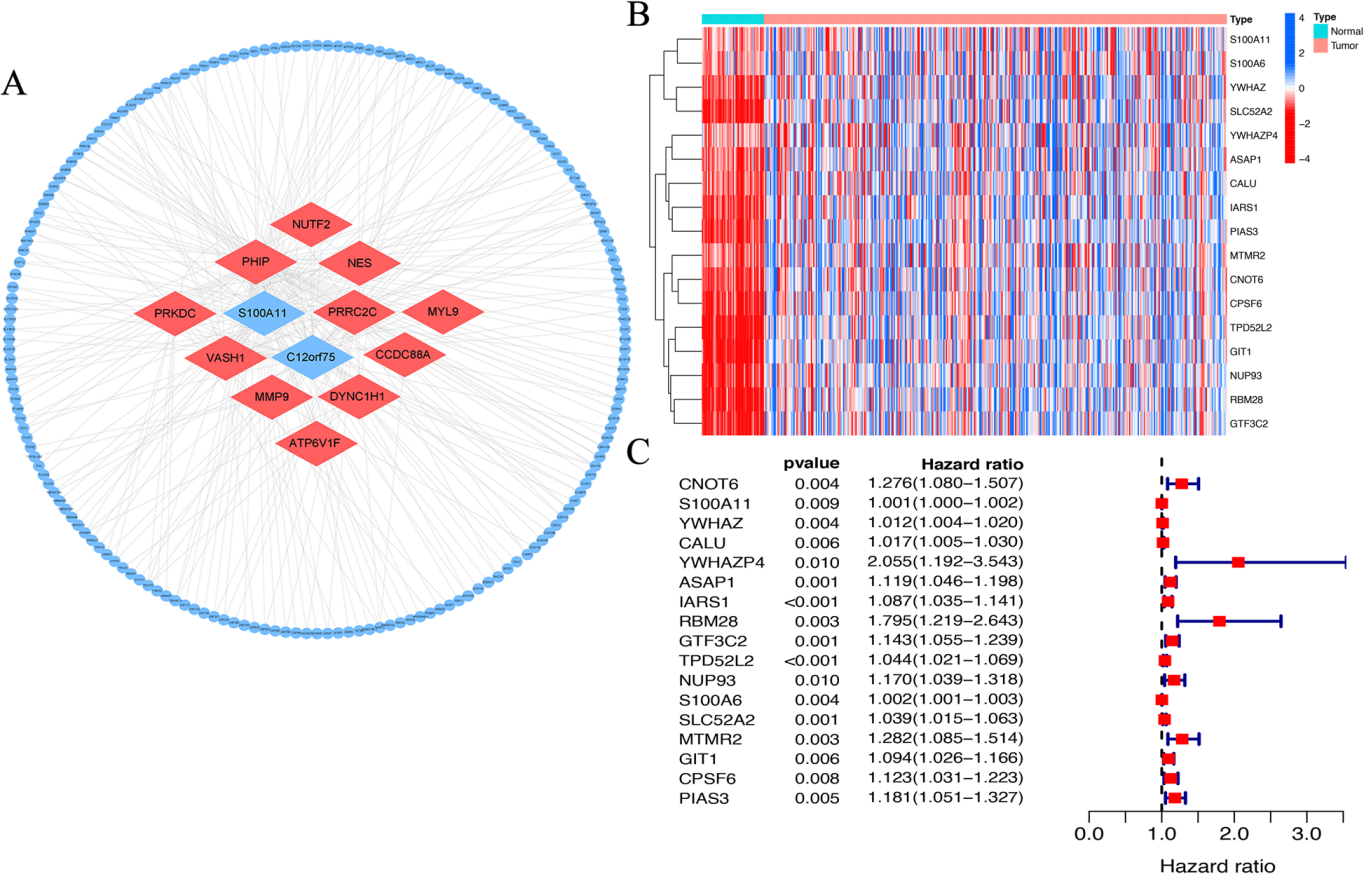
Identification of prognostic ERIGs. **a** Co-expression network of the exosome genes and ERIGs. **b** Heatmap for 17 differentially expressed ERIGs. Red indicats high expression and blue indicates low expression. **c** Forest map for 17 prognostic ERIGs

Correlation analysis between the differential expressed exosome genes and immunosuppressive genes was performed to determine ERIGs. The results showed that there were 13 differentially expressed exosome genes, which co-expressed with 184 immunosuppressive genes (*R* > 0.4, *p* < 0.001) (Fig. 3A). Among 184 ERIGs, 17 were identified as prognostic genes by univariate Cox regression analysis. All the 17 prognostic ERIGs were upregulated in HCC tissues (Fig. 3B). It was found that high expressions of 17 prognostic ERIGs indicated bad prognosis in HCC (Fig. 3C). Subsequently, 5 key prognostic ERIGs (ASAP1, IARS1, GTF3C2, TPD5L2 and SLC52A2) were screened to construct the prognostic signature by Lasso regression analysis. The risk score of each HCC patient was calculated as the followed formula: Risk score = (0.00012 * ASAP1 expression) + (0.03015 * IARS1 expression) + (0.01875 * GTF3C2 expression) + (0.01875 * TPD52L2 expression) + (0.00493 * SLC52A2 expression).

### Validation of the prognostic ERIGs signature

**Fig. 4:**
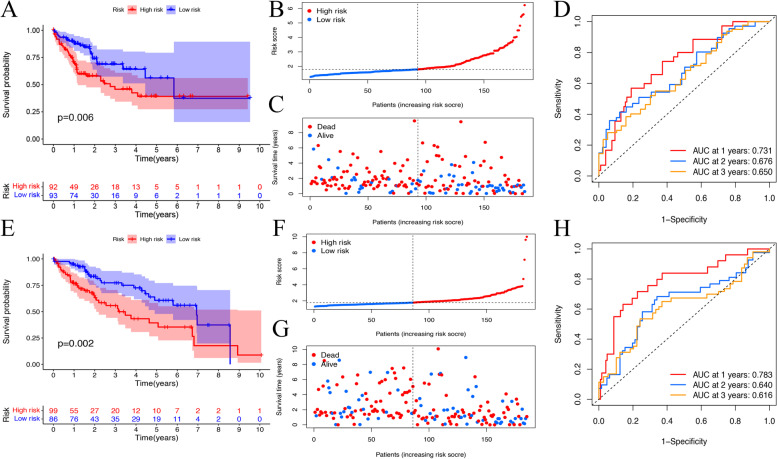
Verification of the ERIGs prognostic signature. **a** Kaplan–Meier survival curves of the OS of patients in the high- and low-risk cohorts for the training cohort. **b** Distribution of patients with different risk scores in the training cohort. **c** OS status of patients with different risk scores in the training cohort. **d** ROC analysis of 1-, 2-, and 3-year in the training cohort. **e** Kaplan–Meier survival curves of the OS of patients in the high- and low-risk cohorts for the validation cohort. **f** Distribution of patients with different risk scores in the validation cohort. **g** OS status of patients with different risk scores in the validation cohort. **h** ROC analysis of 1-, 2-, and 3-year in the validation cohort

HCC patients were classified into the low- and high-risk groups based on the median risk score of the training cohort. Survival analysis revealed that HCC patients in the low-risk group had better prognosis (Fig. 4A). Survival status scatter plots showed that patients in the high-risk group had shorter survival in comparison with those in the low-risk group (Fig. 4B-C). The similar results could be found in the validation cohort, the ICGC cohort and local cohort (Fig. 4E-G, Fig.S1A-C, Fig.S1E-G). ROC curves showed that the signature had a good performance in the prediction of HCC prognosis (Fig. 4D). The AUC reached 0.731 at 1-year, 0.676 at 2-year, and 0.650 at 3-year in the training cohort, respectively, while AUC reached 0.783 at 1-year, 0.640 at 2-year, and 0.616 at 3-year in the validation cohort, respectively (Fig. 4H). In the ICGC cohort and local cohort, our signature also showed a good performance in predicting HCC prognosis (Fig.S1D and Fig.S1H). To further verify the prognostic value of this signature, multivariate Cox regression analysis was performed in the training cohort (Table 1) and validation cohort (Table 2). The results showed that the risk score was an independent predictor of HCC. All these results suggest that this signature has a good performance in predicting HCC prognosis.

**Table 1:** The characteristics of HCC patients in training cohort

Characteristic	HR	95%Cl	*p* value
age	1.033	1.009–1.057	0.006
gender	1.432	0.801–2.561	0.225
grade	0.864	0.585–1.277	0.463
stage	1.390	0.987–1.957	0.060
riskScore	1.636	1.239–2.161	0.001

**Table 2:** The characteristics of HCC patients in validation cohort

Characteristic	HR	95%Cl	*p* value
age	0.997	0.977–1.017	0.744
gender	0.588	0.333–1.037	0.066
grade	1.307	0.851–2.007	0.222
stage	1.763	1.308–2.378	< 0.001
riskScore	1.192	1.023–1.389	0.024

### Nomogram based on the signature for predicting the OS of HCC patients

**Fig. 5:**
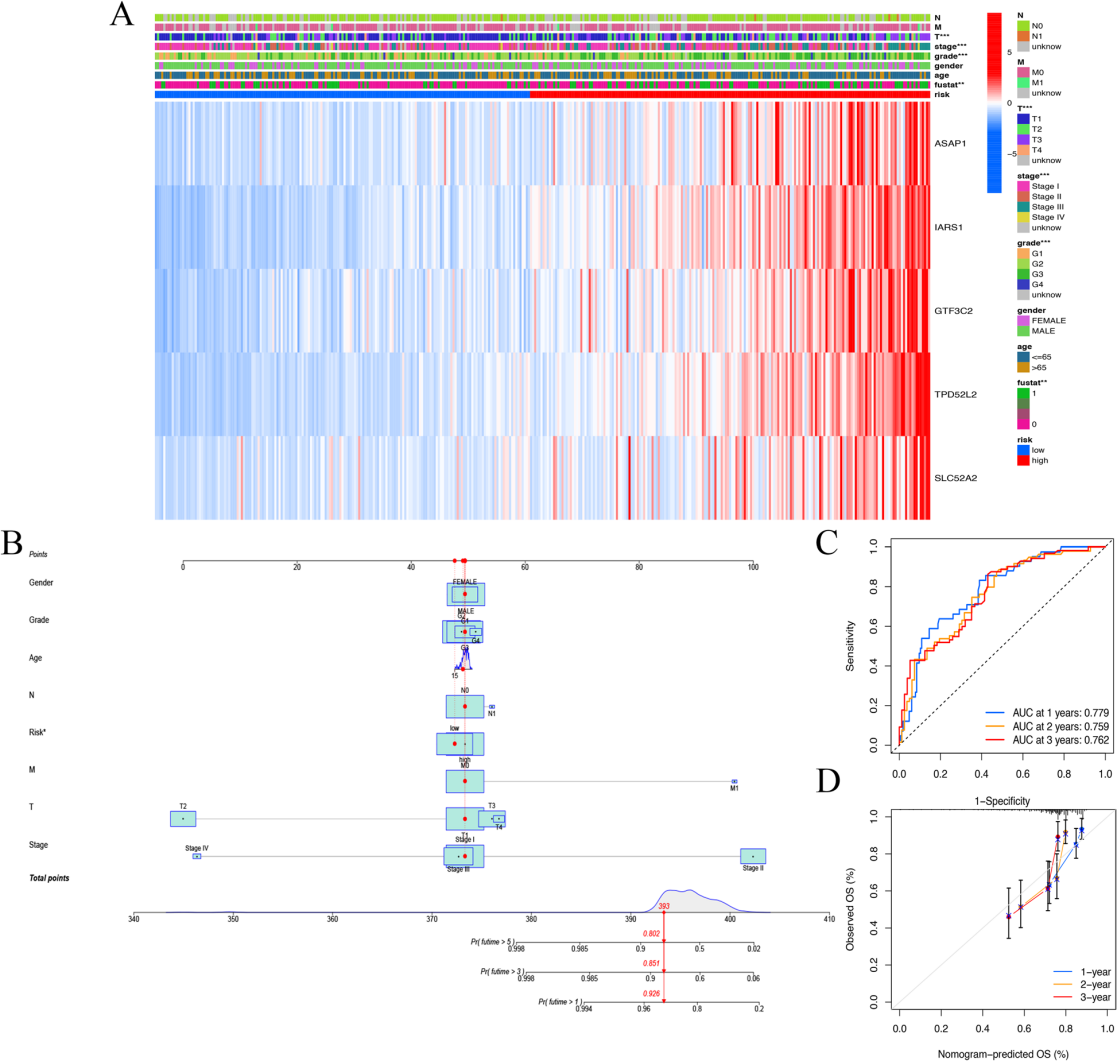
Nomogram based on the signature for predicting the OS of HCC patients. **a** Complex heatmap. **b** The nomogram for the prediction in the probability of patients’ OS. **c** Assessment of prognostic effect of nomogram. **d** Calibration curve was used to evaluate nomogram accuracy

A complex heatmap was plotted to display the relationships among the expressions of signature ERIGs, risk score and clinical characteristics. The result showed that risk score was correlated with T stage, grade, stages and survival status (Fig. 5A). Then, a nomogram, including the risk score and various clinical characteristics (Gender, Grade, Age, T, M, N, Risk), was constructed to better predict HCC prognosis (Fig. 5B). Time-dependent ROC was carried out to investigate the value of the nomogram in predicting the prognosis of HCC patients. The AUC reached 0.779 at 1-year, 0.759 at 2-year, and 0.762 at 3-year, respectively (Fig. 5C). Calibration curves revealed the potential value of the nomogram in predicting HCC prognosis (Fig. 5D).

### Correlation between the signature and tumor microenvironment

**Fig. 6:**
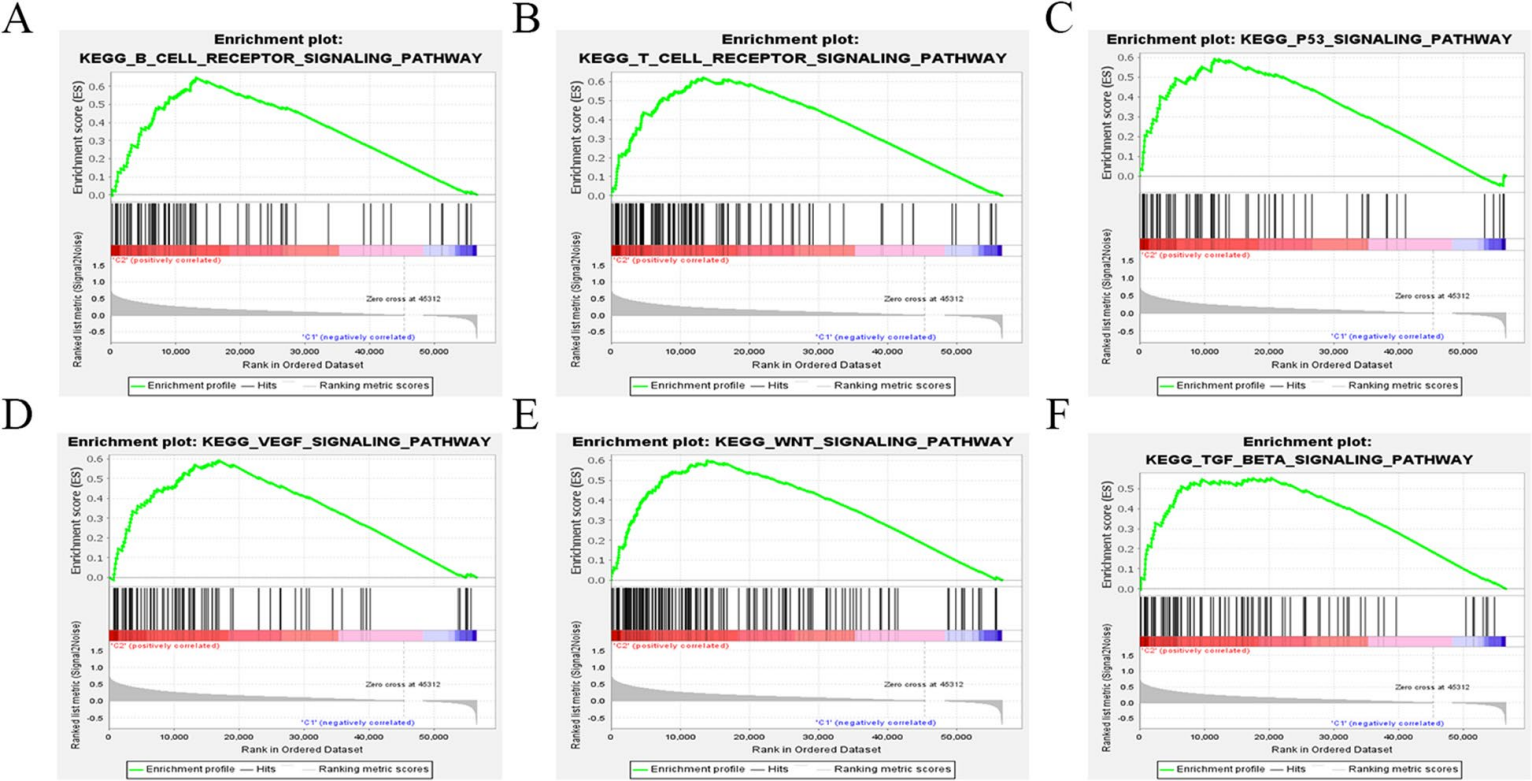
GSEA analysis of the high-risk group. **a** B cell receptor signaling pathway. **b** T cell receptor signaling pathway. **c** P53 signaling pathway. **d** VEGF signaling pathway. **e** WNT signaling pathway. **f** TGFβ signaling pathway

We performed GSEA analysis to explore the biological processes enriched in the high-risk group. Figure 6 showed six significant TME-related pathways that were enriched in high-risk group, including B cell receptor signaling pathway, T cell receptor signaling pathway, P53 signaling pathway, VEGF signaling pathway, WNT signaling pathway, and TGF-beta signaling pathway. These results suggest that these ERIGs of this signature may be involved in the regulation of tumor microenvironment.

**Fig. 7:**
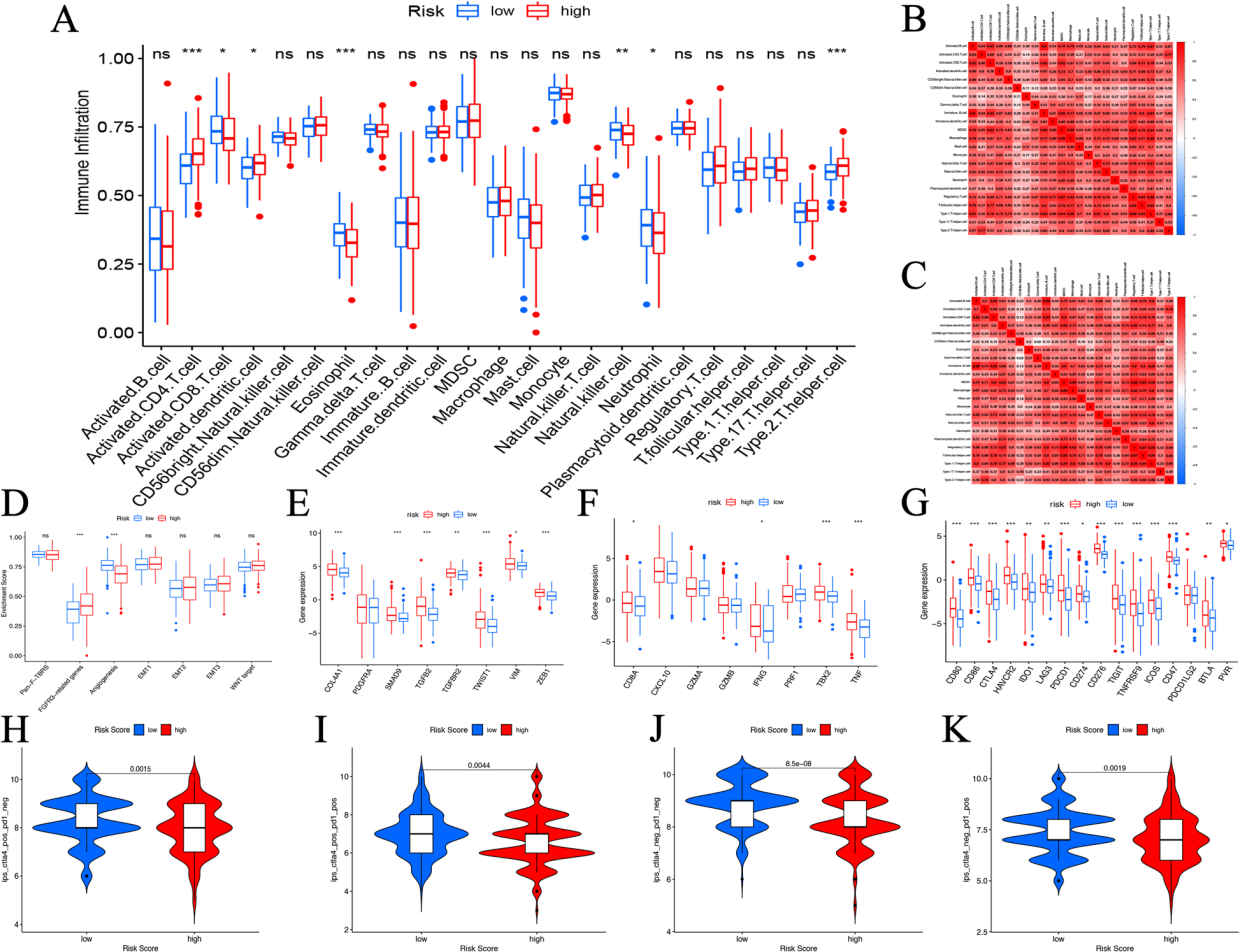
Correlations between the signature and tumor microenvironment. **a** Differences in immune infiltration between the high- and low-risk groups. **b** Correlation matrix of immune cells in the high-risk group. **c** Correlation matrix of immune cells in the low-risk group. **d** Differences in stroma-related pathways between the high- and low- risk groups. **e** Differences in TGF beta- and EMT pathway-related gene expressions between the high- and low-risk groups. **f** Differences in immune-activating gene expressions between the high- and low-risk groups. **g** Differences in immune checkpoint gene expressions between the high- and low-risk groups. **h**-**k** Differences in multiple immunotherapy scores between the high- and low-risk groups

Next, we assessed immune infiltration status in the high- and low-risk groups. Activated CD4 T cell, activated dendritic cell and type 2 T helper cell were highly infiltrated in the high-risk group, while activated CD8 T cell, eosinophil, natural killer cell and neutrophil were upregulated in the low-risk group (Fig. 7A). The correlations among different immune cells were next analyzed in the high- and low-risk groups, respectively. It was found that there were positive correlations among immune cells in both the high- and low-risk groups (Fig. 7B-C). We also analyzed the correlations between stromal-related pathways and this signature. As shown in Fig. 7D, FGFR-related genes were highly expressed in the high-risk group, and angiogenesis pathway was highly expressed in the low-risk group. In addition, TGF-beta and EMT pathway-related genes (COL4A1, SMAD9, TGFB2, TGFBR2, TWIST1, VIM, ZEP1) as well as immune activation genes (CDBA, IFNG, TBX2, TNF) were upregulated in the high-risk group (*p* < 0.05) (Fig. 7E-F). Besides, multiple immune checkpoint genes (CTLA4, PD-1 and PD-L1), which have been widely used as targets for immunotherapy, were found to be highly expressed in the high-risk group (Fig. 7G). Finally, the relationships between the signature and immunotherapy response were also analyzed. In response to immune checkpoints CTLA4 and PD-1, the patients in the low-risk group had higher immunotherapy scores, indicating that patients in the low-risk group had better response to immunotherapy (Fig. 7H-K). Taken together, the above results suggest that our signature may be involved in regulating TME and could serve as an indicator of immunotherapy for HCC.

### The function validation of the five ERIGs in Hep3B cells

To explore the roles of these five ERIGs in HCC proliferation, CCK8 assays were performed in Hep3B cells with knockdown of 5 ERIGs. The results showed that knockdown of the five ERIGs suppressed Hep3B cell proliferation (Fig.S2). Our results suggest that the five ERIGs may be associated with HCC prognosis.

## Discussion

Increasing evidence has indicated that exosomes are involved in cell proliferation, metastasis and drug resistance, and exosomes-related genes have been demonstrated to act as biomarkers of tumors such as HCC [24, 25]. Moreover, exosomes can mediate immunosuppression to aggravate tumor progression [26]. Yang et al. found that nasopharyngeal cancer cell-derived exosomal PD-L1 inhibits CD8+ T-cell activity and promotes immune escape [27]. Xiang et al. confirmed that tumor exosomes induce myeloid-derived suppressor cells to promote tumor progression [28]. From the perspective of clinical application, exosomes can be regarded as the potential targets for cancer diagnosis, prognosis and therapy. However, the roles of ERIGs in tumor progression are still largely unknown. This is a first report to focus on ERIGs, which comprehensively investigated prognostic ERIGs in HCC. Lasso regression analysis was used to construct a 5-ERIG signature (ASAP1, IARS1, GTF3C2, TPD5L2 and SLC52A2) to predict HCC prognosis. Then we validated the efficacy of the prognostic signature using a variety of methods. Subsequent functional analysis revealed the correlations between the signature and TME-related pathways as well as molecules, especially immune-related pathways and immune checkpoints. All the results suggest that our prognostic signature could effectively predict HCC prognosis and may guide individualized immunotherapy.

Several studies have shown that ERIGs play an important role in HCC metastasis, drug resistance and immune escape [29–31]. Yang et al. reported that immune cells- derived exosomes containing integrin CD147 may facilitate HCC metastasis [32]. Lv et al. found that exosomes carrying HSP secreted by hepatocellular cancer cells under stress conditions can enhance immunogenicity and induce natural killer cell response [33]. Moreover, HMGB1 has been reported to promote the amplification of TIM-1 + regulatory B cells to promote HCC immune escape [34]. In particular, ASAP1 and TPD52L2, members of our signature, have been found to promote the proliferation and invasion of HCC [35–37]. ASAP1 has also been reported to promote HCC metastasis and mediate tumor-associated macrophage infiltration in HCC [38]. In addition, knockdown of TPD52L2 can inhibit the proliferation of HCC cells in vitro, suggesting that TPD52L2 may be a potential target for the diagnosis and treatment of HCC [36]. GTF3C2 may serve as a potential prognostic marker to predict HCC prognosis [39]. However, the functions of IARS1 and SLC52A2 in HCC progression remain to be explored in the future. Taken together, the 5 ERIGs may participate in the regulation of HCC progression and further experiments are needed in the future.

Notably, the relationships between the prognostic signature and many characteristic pathways or molecules of TME were also explored. A variety of immune cells were found to be down-regulated in the high-risk group, including activated CD8 T cell, eosinophil, natural killer cell and neutrophil. Meanwhile, TGF-beta and EMT pathway-related genes were shown to be upregulated in the high-risk group. These results suggest that 5 ERIGs of this signature may affect tumor progression by regulating the expression of the above immune cells and TGF-beta and EMT pathway-related genes. Moreover, the expressions of immune checkpoints were highly expressed in the high-risk group and immunotherapy scores were significantly higher in the low-risk group. Recently, monoclonal antibody medicines targeting PD-1 (atezolizumab) or PD-L1 (pembrolizumab) have been used for HCC with satisfactory therapeutic effect in a number of patients [40]. In this study, immune checkpoint genes such as PD-1 (PDCD1) and PD-L1 (CD274) were found to be upregulated in the high-risk group. Combine with these, our findings may provide many guidance for immunotherapy of HCC.

This study has some limitations. Firstly, more HCC clinical cohorts are needed to validate our signature. Secondly, the relationship between the identified immunosuppressive genes and exosomes requires experimental validation. Finally, in vitro and in vivo experiments are required to investigate the underlying immune-related mechanisms of these immunosuppressive genes.

## Conclusion

In conclusion, we identified a novel signature based on ERIGs, which may provide a personalized prediction for the prognosis of HCC patients and immunotherapy response.

## Supplementary Information

**Additional file 1 **: **Fig. S1** Verification of the ERIGs prognostic signature in the ICGC cohort and local cohort. A Kaplan–Meier survival curves of the OS of patients in the high- and low-risk cohorts for the ICGC. B Distribution of patients with different risk scores in the ICGC cohort. C OS status of patients with different risk scores in the ICGC cohort. D ROC analysis of 1-, 2-, and 3-year in the ICGC cohort. E Kaplan–Meier survival curves of the OS of patients in the high- and low-risk cohorts for the local cohort. F Distribution of patients with different risk scores in the local cohort. G OS status of patients with different risk scores in the local cohort. H ROC analysis of 1-, 2-, and 3-year in the local cohort.

**Additional file 2 **: **Fig. S2** The function validation of the five ERIGs in Hep3B cell using CCK8. *p < 0.05. Data are presented as mean ± SD of at least three independent experiments.

## Data Availability

The datasets generated and/or analyzed during the current study are available in the exoRbase repository (http://www.exorbase.org/), TCGA repository (http://cancergenome.nih.gov/ and ICGC repository (https://dcc.icgc.org/).
