# Supplementary figures and images for: A comprehensive study based on exosome-related immunosuppression genes and tumor microenvironment in hepatocellular carcinoma
Source: BMC Cancer. 2022 Dec 22;22:1344. doi: 10.1186/s12885-022-10463-0 (PMC9773453; doi:10.1186/s12885-022-10463-0)

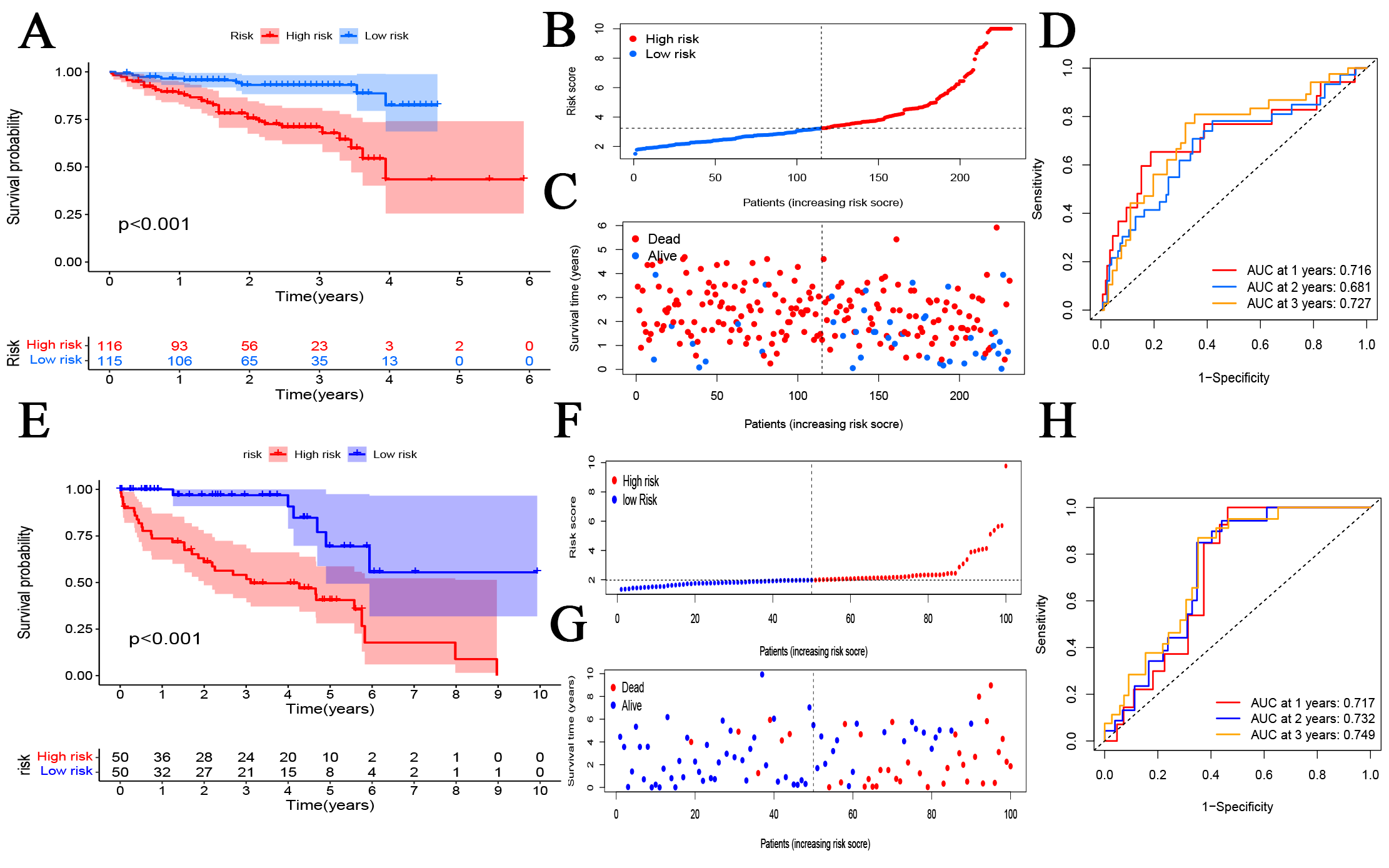

Supplement: Supplementary file 1 — Additional file 1 : Fig. S1 Verification of the ERIGs prognostic signature in the ICGC cohort and local cohort. A Kaplan–Meier survival curves of the OS of patients in the high- and low-risk cohorts for the ICGC. B Distribution of patients with different risk scores in the ICGC cohort. C OS status of patients with different risk scores in the ICGC cohort. D ROC analysis of 1-, 2-, and 3-year in the ICGC cohort. E Kaplan–Meier survival curves of the OS of patients in the high- and low-risk cohorts for the local cohort. F Distribution of patients with different risk scores in the local cohort. G OS status of patients with different risk scores in the local cohort. H ROC analysis of 1-, 2-, and 3-year in the local cohort. [file 12885_2022_10463_MOESM1_ESM.tif]

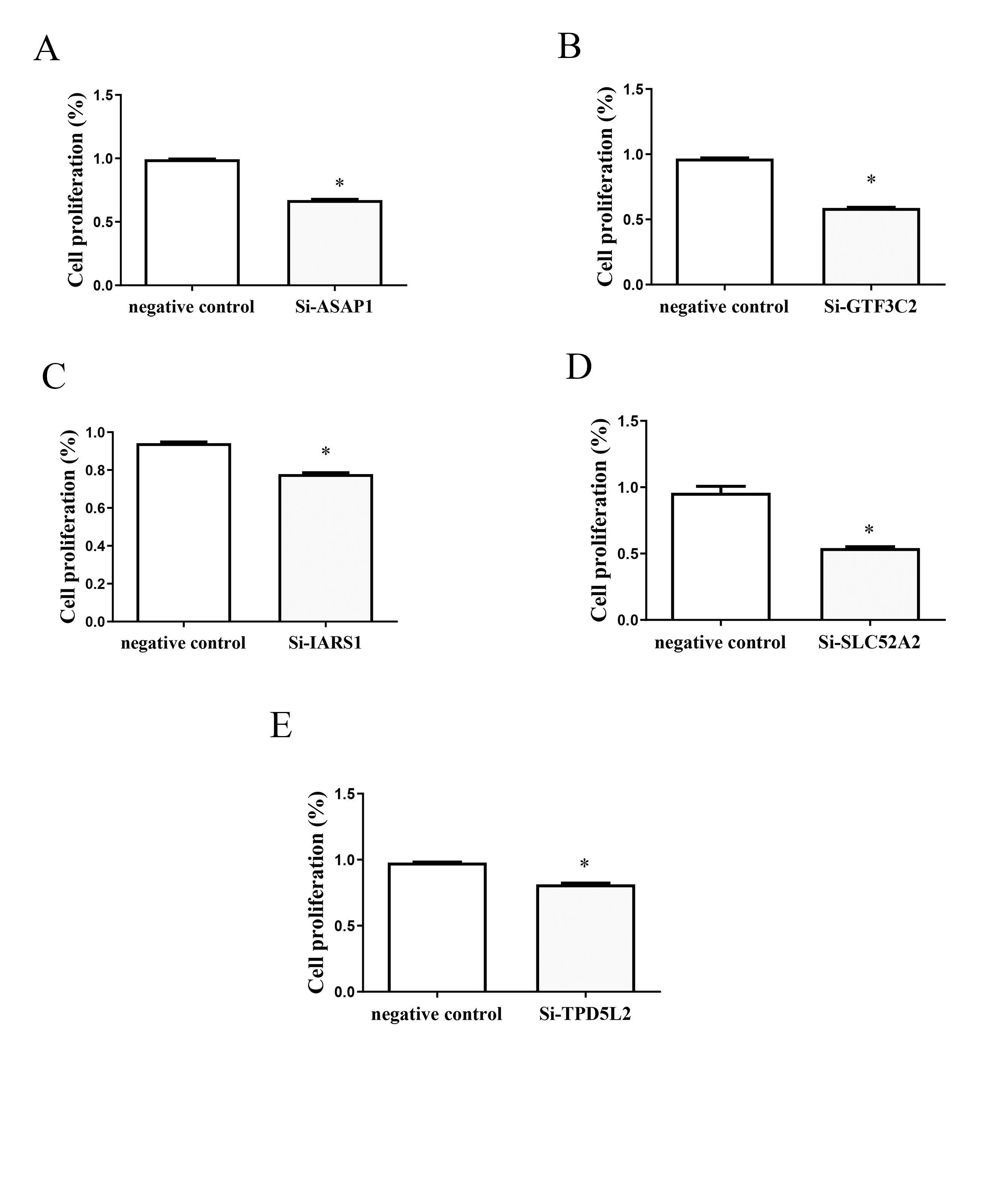

Supplement: Supplementary file 2 — Additional file 2 : Fig. S2 The function validation of the five ERIGs in Hep3B cell using CCK8. *p < 0.05. Data are presented as mean ± SD of at least three independent experiments. [file 12885_2022_10463_MOESM2_ESM.tif]
